# Are scale-free networks robust to measurement errors?

**DOI:** 10.1186/1471-2105-6-119

**Published:** 2005-05-16

**Authors:** Nan Lin, Hongyu Zhao

**Affiliations:** 1 Department of Mathematics, Washington University in St. Louis, St. Louis, MO 63143, USA; 2 Department of Epidemiology and Public Health, Yale University, New Haven, CT 06520, USA; 3 Department of Genetics, Yale University, New Haven, CT 06520, USA

## Abstract

**Background:**

Many complex random networks have been found to be scale-free. Existing literature on scale-free networks has rarely considered potential false positive and false negative links in the observed networks, especially in biological networks inferred from high-throughput experiments. Therefore, it is important to study the impact of these measurement errors on the topology of the observed networks.

**Results:**

This article addresses the impact of erroneous links on network topological inference and explores possible error mechanisms for scale-free networks with an emphasis on *Saccharomyces cerevisiae *protein interaction networks. We study this issue by both theoretical derivations and simulations. We show that the ignorance of erroneous links in network analysis may lead to biased estimates of the scale parameter and recommend robust estimators in such scenarios. Possible error mechanisms of yeast protein interaction networks are explored by comparisons between real data and simulated data.

**Conclusion:**

Our studies show that, in the presence of erroneous links, the connectivity distribution of scale-free networks is still scale-free for the middle range connectivities, but can be greatly distorted for low and high connecitivities. It is more appropriate to use robust estimators such as the least trimmed mean squares estimator to estimate the scale parameter *γ *under such circumstances. Moreover, we show by simulation studies that the scale-free property is robust to some error mechanisms but untenable to others. The simulation results also suggest that different error mechanisms may be operating in the yeast protein interaction networks produced from different data sources. In the MIPS gold standard protein interaction data, there appears to be a high rate of false negative links, and the false negative and false positive rates are more or less constant across proteins with different connectivities. However, the error mechanism of yeast two-hybrid data may be very different, where the overall false negative rate is low and the false negative rates tend to be higher for links involving proteins with more interacting partners.

## Background

Recent studies have found that many complex networks, ranging from the World-Wide Web [1] and the scientific collaboration network [2] to biological systems such as the yeast protein interaction network [3], are scale-free. The scale-free property states that the distribution of the connectivity *k *(number of links per node) in a network can be described by the power law, i.e.,

*P*(*k*) = *ck*^-*γ*^, *c *> 0, *γ *> 0.     (1)

A visual diagnosis of the scale-free behavior can be made through the log-log plot of the connectivity distribution, in which a straight line with slope -*γ *is expected. In scale-free networks, the nodes are not randomly or evenly connected with some highly connected nodes ("hubs"). The ratio of the number of "hubs" to that of nodes in the rest of the network remains constant as the network changes in size. One attractive feature is that scale-free networks are more resistant to random failures compared with random networks due to the existence of a few highly connected "hubs" [4]. Remarkably, it has been observed that the scale parameter *γ *varied only in the narrow range of 2.1 – 4 in the aforementioned real-world networks. All existing studies on scale-free networks assumed that the observed links represented the underlying structure of the network, but paid little attention to the fact that the observed links often involved errors, namely, false positives and false negatives. For example, Jeong *et al*. [3] considered the *Saccharomyces cerevisiae *protein interaction network inferred from yeast two-hybrid (Y2H) experiments. It is well-known that the Y2H system has many false positives as well as false negatives [5]. A natural question to ask is whether a scale-free network is still observed as scale-free in the presence of errors. And if it is, what are the possible underlying error mechanisms and how variable is the observed scale parameter *γ*? Answering these questions may lead to further insight to the scale-free property, better understanding and correct usage of the observed network data. For convenience, we will call networks observed with erroneous links as perturbed networks in the rest of this article.

## Results

In this article, we address the above questions by both theoretical derivations and simulation studies using the yeast protein interaction network as a prototype. However, the results apply to general scale-free networks.

### Connectivity distribution of scale-free networks with erroneous links under a simple model

We first study how the connectivity distribution of a scale-free network is affected when errors are present. Following previous studies on the reliability of protein interaction networks [6], we assume a simple error mechanism in which the false positive rate (*r*_*FP*_) and false negative rate (*r*_*FN*_) are the same for all node pairs, and false positives and false negatives are independently generated. The false positive rate and false negative rate of a node pair refer to the probability that the pair of nodes is observed as linked when they are actually not and the probability that the pair of nodes is observed as unlinked when they are actually linked. Under this assumption, every truly linked pair of nodes has a probability *r*_*FN *_to be observed as unlinked nodes, and every truly unlinked pair of nodes has a probability *r*_*FP *_to be observed as linked nodes.

The above assumption is similar to the grand canonical ensembles of random networks in Chapter 4 of Dorogovtsev and Mendes [7], in which networks evolve by removing existing edges and adding new edges with certain probabilities. We can also view the perturbed network as obtained by removing edges (false negative) and adding edges (false positive) from the underlying network. The probability of adding an edge between two non-linked nodes is the false positive rate *r*_*FP*_, and the probability of removing the edge between two linked nodes is the false negative rate *r*_*FN*_. However, while Dorogovtsev and Mendes mostly discussed the connectivity distribution of equilibrium networks (networks obtained after infinite times edge adding and removing), we focus on the connectivity distribution of the observed network that are obtained by considering removing every existing edge and adding non-existing edges just once.

#### Connectivity distribution of the perturbed network

In the following, we will derive the distribution of the observed connectivities for a scale-free network of size *n *for given values of *r*_*FP *_and *r*_*FN*_. Let *N*_*P *_and *N*_*T *_denote the observed and true connectivity of a node, respectively. Then the probability to observe a node with *k *links is



The minimum and maximum connectivity of a node, *T*_*min *_and *T*_*max*_, are assumed to be the same for all the nodes in the network, and their values depend on the specific network. In general, we set *T*_*min *_= 0 and *T*_*max *_= *n *- 1 when expert knowledge is not available, where *n *denotes the size of the network, i.e., the total number of nodes in the network. The following elucidates how to calculate (2) analytically. Let *N*_*FP*_, *N*_*TP*_, *N*_*FN*_, *N*_*TN*_, and *N*_*N *_be the numbers of false positive links (observed as linked but actually not), true positive links (observed as linked and actually linked), false negative links (observed as unlinked but actually linked), true negative (observed as unlinked and actually unlinked) and negative links (actually unlinked) associated with the node, respectively. Since the observed links of a node consist of both false positive and true positive ones, and the true links consist of true positive and false negative ones, we have *N*_*P *_= *N*_*FP *_+ *N*_*TP*_, *N*_*T *_= *N*_*FN *_+ *N*_*TP*_, *N*_*N *_= *N*_*FP *_+ *N*_*TN*_, and *T*_*max *_= *N*_*T *_+ *N*_*N*_. Furthermore, underour assumed error mechanism, following similar derivations as shown in [7], *N*_*FP *_and *N*_*FN *_follow the binomial distributions *Bin*(*T*_*max *_- *N*_*T*_, *r*_*FP*_) and *Bin*(*N*_*T*_, *r*_*FN*_), respectively, for a given value of *N*_*T*_. This implies that *r*_*FP *_= *E*(*N*_*FP*_)/(*T*_*max *_- *N*_*T*_) = *E*(*N*_*FP*_)/(*N*_*FP *_+ *N*_*TN*_) and *r*_*FP *_= *E*(*N*_*FN*_)/*N*_*T *_= *E*(*N*_*FN*_)/(*N*_*TP *_+ *N*_*FN*_), where *E*(*X*) denotes the expectation of random variable *X*. Then the conditional probability *P*(*N*_*P *_= *k*|*N*_*T *_= *j*) in (2) can be written as follows.



where *dBin*(*k*; *p*, *n*) = *P*(*X *= *k*) with *X *~ *Bin*(*n*, *p*). Moreover, the power law of the scale-free network implies that *P*(*N*_*T *_= *j*) = *cj*^-*γ*^. Hence, the observed connectivity distribution can be calculated by



#### Simulations

We next explore the impact of the erroneous links on the topology of the scale-free networks. With an emphasis on the yeast protein interaction network, we compute the distribution of the observed connectivity of scale-free networks with the false positive rate (*r*_*FP*_) and false negative rate (*r*_*FN*_) similar to the yeast protein interaction network under the assumption of the aforementioned simple error mechanism. We set the scale parameter *γ *= 3, the size of the network *n *= 1000 or 7000, and vary *r*_*FP *_from 0.0001 to 0.0003 and *r*_*FN *_from 0.1 to 0.9 on 9 equally spaced values. These ranges of *r*_*FP *_and *r*_*FN *_are based on Deng *et al*. [8], in which the authors estimated the false positive rate and false negative rate to be less than 0.000285 and greater than 0.64, respectively, based on the Y2H data. We consider a larger range of *r*_*FP *_to cover other data sources, such as the MIPS complex data, where false positives are less frequent. In the calculations, we use *T*_*min *_= 1 and *T*_*max *_= *n *- 1.

**Figure 1:**
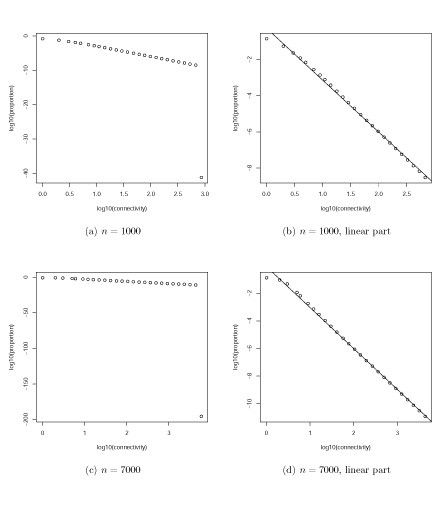
**Connectivity distribution of the perturbed scale-free networks (*r*_*FP *_= 0.0001, *r*_*FN *_= 0.3)**. This picture shows the connectivity distribution of the the perturbed networks using (3) provided that *r*_*FP *_= 0.0001 and *r*_*FN *_= 0.3. Figure 1(b) and 1(d) are the linear parts of Figure 1(a) and 1(c), respectively, imposed with the regression lines fitted by the OLS.

**Figure 2:**
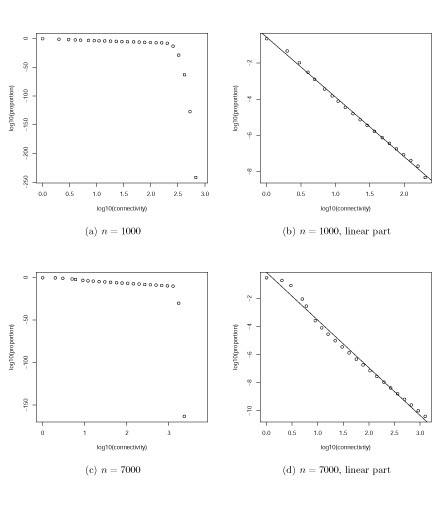
**Connectivity distribution of the perturbed scale-free networks (*r*_*FP *_= 0.00015, *r*_*FN *_= 0.8)**. This picture shows the connectivity distribution of the the perturbed networks using (3) provided that *r*_*FP *_= 0.00015 and *r*_*FN *_= 0.8. Figure 2(b) and 2(d) are the linear parts of Figure 2(a) and 2(c), respectively, imposed with the regression lines fitted by the OLS.

In the log-log plot (Figures 1 and 2) of the observed connectivity distribution of the perturbed networks when (*r*_*FP *_= 0.0001, *r*_*FN *_= 0.3) and (*r*_*FP *_= 0.00015, *r*_*FN *_= 0.8), it can be seen that the connectivity distribution after perturbation still maintains the scale-free property in the middle range of the connectivity, but deviates from the original linear pattern at both the small and large connectivity regions. The slope of the linear part is close to the true value -3 (see Tables A.1 and A.2 in Additional file 1). The deviation is more significant in the large connectivity region than that in the small connectivity region. This deviation pattern is consistent across networks of different sizes considered in our calculations (data not shown). Comparisons among the observed connectivity distributions (figures not shown) of perturbed networks with different values of r_*FP *_and *r*_*FN *_suggest that the deviation depends little on *r*_*FP *_but largely on *r*_*FN*_. As *r*_*FN *_increases, the deviation of the tail probability becomes more significant. This deviation is also more obvious in a smaller network.

#### Estimation of *γ*

The connectivity distribution of the perturbed network suggests a cautious use of the observed link data, especially on estimating *γ*. The scaling parameter *γ*, an important characteristic measure of the scale-free network, is commonly estimated using the ordinary least squares (OLS) in the linear model from the log transformation of (1).

log *P*(*k*) = log *c *- *γ *log *k*.     (4)

It is well known that the OLS estimator can be very sensitive to even a small number of outliers. For example, applying the OLS estimator in Figure 1(a) will not be able to capture the linear trend if the point at the last end is included in the estimation. Therefore, robust estimators, such as the M-estimator and the least trimmed squares (LTS) estimator [9] are more proper choices in such situations due to their resistance to outliers. Our simulations suggest that the LTS estimator can correctly capture the linear trend without visual diagnosis of the connectivity distribution, while the OLS and M-estimator often fail to estimate the slope of the linear part correctly. Therefore, we will use the LTS estimator in our following simulation studies.

### Exploring error mechanisms of yeast protein interaction networks by simulations

In the previous section, we found that the scale-free property can be conserved to a large extent under a simple error mechanism. However, the error mechanisms of the real data are often more complicated. For more complicated error mechanisms, theoretical derivations of the connectivity distribution of the perturbed networks are often intractable. But it is also important to know how the empirical connectivity distributions of real networks are affected by the erroneous links. Therefore, we conduct extensive simulation studies to investigate the finite-sample impact of the error mechanisms on the connectivity distribution. Our study focuses on the yeast protein-interaction network data.

**Figure 3:**
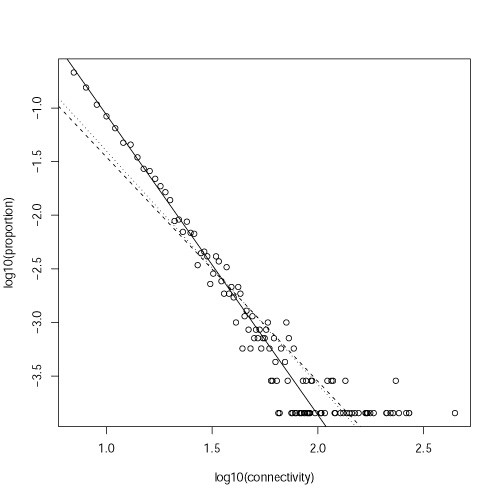
**Connectivity distribution of *Net*_0_**. This picture shows the connectivity distribution of the simulated scale-free random network *Net*_0 _imposed with regression lines given by different methods (dashed line: OLS; dotted line: M-estimation; solid line: LTS).

**Figure 4:**
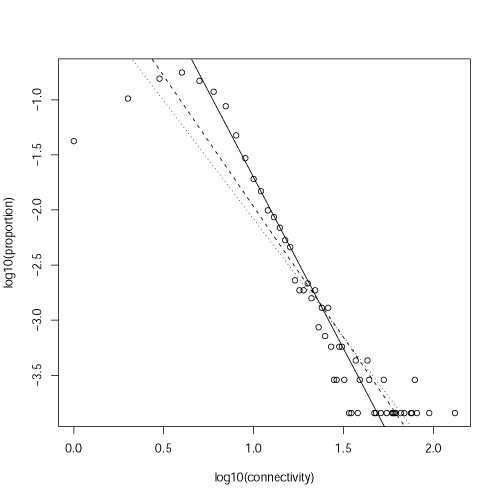
**Connectivity distribution of *Net*_0 _after perturbation (*r*_*FP *_= 0.0002, *r*_*FN *_= 0.7)**. This picture shows the connectivity distribution of the simulated scale-free random network *Net*_0 _perturbed by the simple error mechanism using *r*_*FP *_= 0.0002 and *r*_*FN *_= 0.7. Regression lines given by different methods are also imposed (dashed line: OLS; dotted line: M-estimation; solid line: LTS).

For real network data, no matter whether erroneous links are involved or not, the empirical connectivity distribution will not display a linear pattern as clear as the ones in Figure 1 due to sampling variations and its discrete approximation to the tiny probability of nodes with large connectivities. For example, Figure 3 shows the connectivity distribution of a simulated scale-free network *Net*_0 _and Figure 4 shows the connectivity distribution of *Net*_0 _after perturbation by the simple error mechanism discussed above. In Figure 4, we observe a much larger curvature deviation from the linear trend at the small connectivity region than that in Figures 1 and 2. It is not clear why the empirical distributions of the simulated networks are so different from the theoretical calculations, but this observation demonstrates that simulation studies are necessary to complement the findings from the theoretical calculations. In addition, simulation studies can also explore possible error mechanisms by comparing the connectivity distributions of simulated perturbed scale-free networks with the observed networks by assuming that their underlying structure are indeed scale-free.

In the following, we investigate the error mechanisms of two real yeast protein interaction network data sets used in Jeong *et al*. [3] and Deng *et al*. [6] by comparing the connectivity distribution of these two networks with that of the simulated network perturbed by different error mechanisms. We assume that the true underlying topology of the yeast protein interaction network is scale-free [3]. Then if we perturb the simulated scale-free network by the error mechanisms similar to the ones of the real yeast protein interaction networks, the resulting connectivity distribution should be similar to the ones of the real networks.

#### MIPS and Y2H yeast protein networks

**Figure 5:**
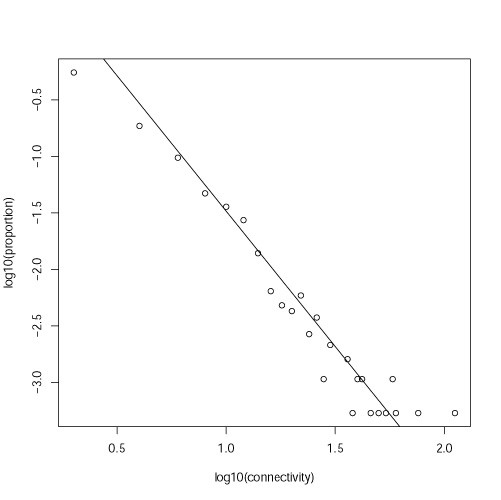
**Connectivity distribution of the Y2H yeast protein interaction network**. This picture shows the connectivity distribution of the protein interaction network in Jeong *et al*. [3] inferred from the Y2H data. The imposed regression line is fitted by the LTS method.

**Figure 6:**
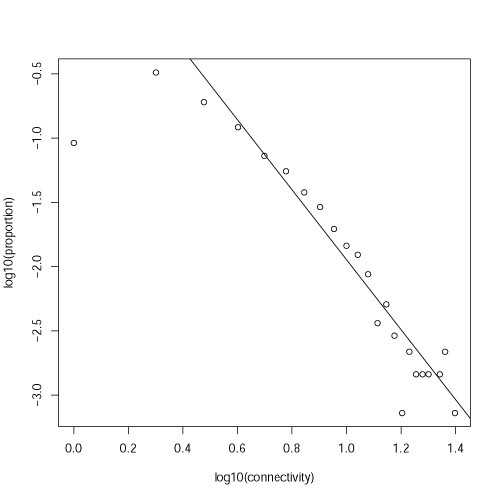
**Connectivity distribution of the MIPS yeast protein interaction network**. This picture shows the connectivity distribution of the protein interaction network in Deng *et al*. [6] inferred from the MIPS gold standard data. The imposed regression line is fitted by the LTS method.

Jeong *et al*. derived the yeast protein network from combined, non-overlapping Y2H data [10,11]. This network has 1,870 proteins as nodes, connected by 2,240 identified direct physical interactions [12]. The other network was obtained from the gold standard of yeast protein interactions based on the MIPS complex data [13]. This gold standard data set has 1,376 proteins and 2,876 interacting protein pairs, out of which 2,559 are also recorded in the Yeast Proteome Database (YPD) [14]. The YPD subset has 1,373 proteins. Estimates of *γ *from the Y2H network, the gold standard data and the YPD subset are 2.396, 2.721 and 2.870, respectively. The connectivity distributions of these two networks are shown in Figure 5 and Figure 6, respectively.

#### Error mechanisms

We consider different error mechanisms in terms of different types of false positive rates (*p*_*ij *_= *P *(*x*_*i *_and *x*_*j *_are observed linked|*x*_*i *_and *x*_*j *_are actually unlinked)) and false negative rates (*q*_*ij *_= *P *(*x*_*i *_and *x*_*j *_are observed unlinked|*x*_*i *_and *x*_*j *_are actually linked)) for node pair (*x*_*i*_, *x*_*j*_), *i *= 1,..., *n*, *j *= 1,..., *n*, *i *≠ *j*. Assume that the overall false positive rate and false negative rate are *r*_*FP *_and *r*_*FN*_, in the sense that the expected number of false positive links and false negative links are *E*(*N*_*FP*_) = *r*_*FP *_*N*_*N *_and *E*(*N*_*FN*_) = *r*_*FN *_*N*_*P*_. We consider nine different error mechanisms by letting *p*_*ij *_and *q*_*ij *_be one of the following three different types:

1. **constant**: *p*_*ij *_= *r*_*FP *_and *q*_*ij *_= *r*_*FN *_for all (*x*_*i*_, *x*_*j*_);

2. **increasing (with connectivity)**:



3. **decreasing (with connectivity)**:



where *L*(*x*) denotes the true connectivity of node *x*. For *Net*_0_, *N*_*P *_= 49, 007 and *N*_*N *_= 24, 503, 521. The combinations of different structures on false positive rates and false negative rates produce nine error mechanisms in Table 1.

#### Simulation studies

We simulate a scale-free network *Net*_0 _using the preferential attachment growth model [15,16]. In this algorithm, we start from *m*_0 _= 7 isolated nodes and add *m *= 7 links to the existing nodes with probability proportional to their connectivity in each of the *T *= 7, 000 evolving steps. *Net*_0 _has *L *= 49, 007 links and *n *= 7, 008 nodes. The mean-field theory [15] suggests that the theoretical value of *γ *for *Net*_0 _is 3, which agrees well with the estimates in Table 2.

**Figure 7:**
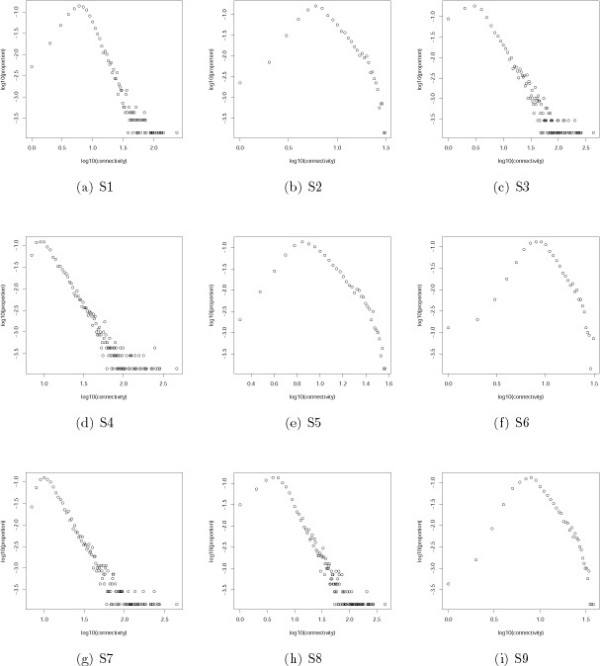
Connectivity distribution of *Net*_0 _perturbed by different error mechanisms (*r*_*FP *_= 0.00025, *r*_*FN *_= 0.5).

**Figure 8:**
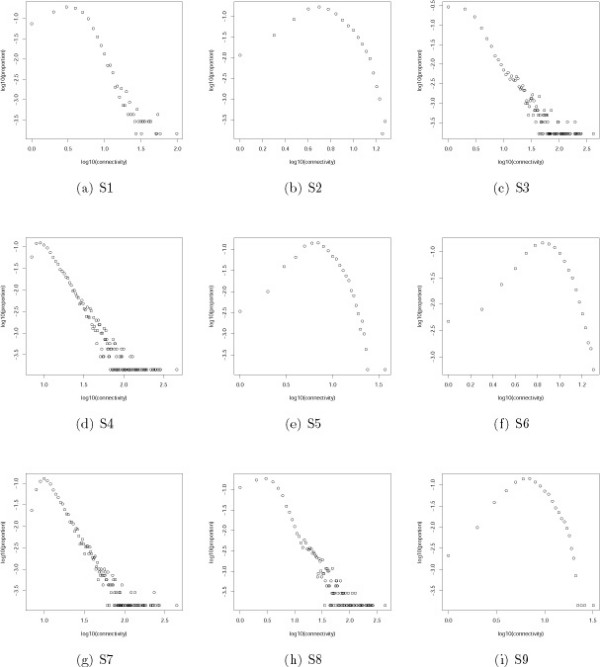
Connectivity distribution of *Net*_0 _perturbed by different error mechanisms (*r*_*FP *_= 0.00025, *r*_*FN *_= 0.8).

**Figure 9:**
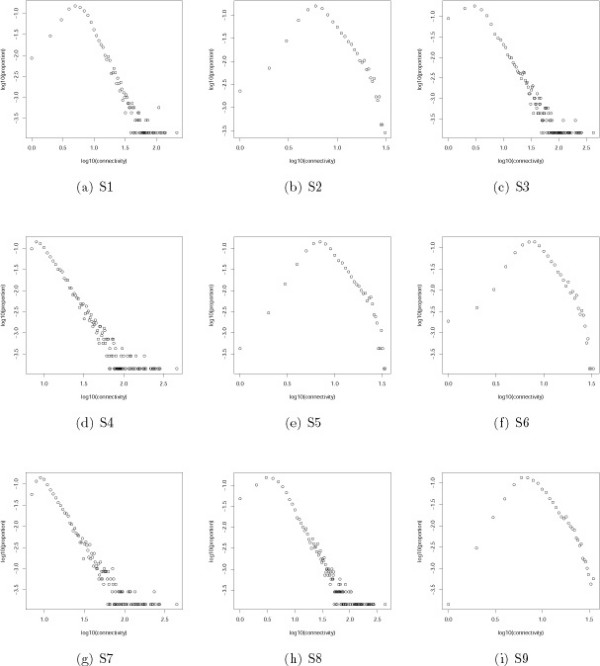
Connectivity distribution of *Net*_0 _perturbed by different error mechanisms (*r*_*FP *_= 0.00015, *r*_*FN *_= 0.5).

**Figure 10:**
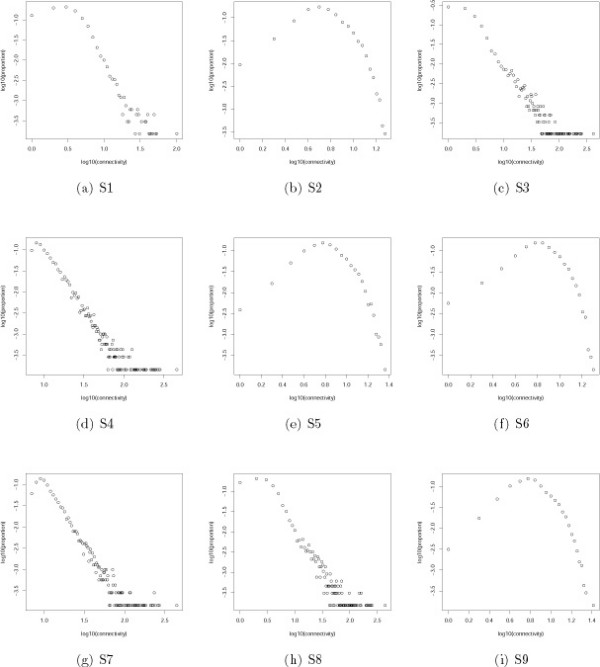
Connectivity distribution of *Net*_0 _perturbed by different error mechanisms (*r*_*FP *_= 0.00015, *r*_*FN *_= 0.8).

We always assume that false positives and false negatives are independently generated. In the simulations, a link is added (false positive) between every two unlinked nodes (*x*_*i*_, *x*_*j*_) in *Net*_0 _with probability *p*_*ij*_, and the link is removed (false negative) between two linked nodes (*x*_*i*_, *x*_*j*_) in *Net*_0 _with probability *q*_*ij*_. We also consider these error mechanisms under high and low overall false positive (*r*_*FP*_) and false negative rates (*r*_*FN*_). The connectivity distributions of *Net*_0 _after perturbation are shown in Figures 7, 8, 9, 10 for different values of *r*_*FP *_and *r*_*FN*_: (0.00025, 0.5), (0.00025, 0.8), (0.00015, 0.5), (0.00025, 0.8).

Under the nine different error mechanisms, the connectivity distribution of the perturbed *Net*_0 _can be dramatically different. Under error mechanisms *S*2, *S*5, *S*6 and *S*9, the perturbed networks contain a small proportion of nodes with low connectivity, which differs greatly from the observed yeast protein interaction networks (Figures 5 and 6). This finding suggests that these four mechanisms are far different from the true error structure, and we will not discuss them in the following. We also observe that changes in *r*_*FP *_render little impact on the connectivity distribution under all error mechanisms, but a higher value of *r*_*FN *_increases the probability of nodes with small connectivity under *S*1, *S*3 and *S*8. And mechanisms *S*4 and *S*7 are highly stable structures, that is, the connectivity distribution changes little in response to changes in *r*_*FP *_or *r*_*FN *_under these two error mechanisms. This suggests that scale-free networks with constant false negative rates can still provide very credible information about its topological structure. This finding is also confirmed by the fact that the estimates of *γ *vary little when *r*_*FN *_changes (see Tables A.5 and A.6 in Additional file 1). The estimated values of *γ *vary only from 2.61 to 3.03 with a standard error of 0.125 under *S*4 and only from 2.56 to 3.31 with a standard error of 0.161 under *S*7, whereas the estimate of *γ *clearly decreases as *r*_*FN *_increases under *S*3 and *S*8 (Tables A. 4 and A. 7 in Additional file 1). Under *S*1, there is no clear pattern on the estimated *γ *as *r*_*FN *_changes (Table A.3 in Additional file 1), but the estimates of *γ *vary in a much wider range (1.16 – 4.35) compared with those under *S*3 and *S*8. It is worth noting that our conclusions are restricted to the particular range of *r*_*FP *_and *r*_*FN *_we have studied, however these ranges are believed to be reasonable to describe the Y2H systems.

The simple error mechanism *S*1 with a high false negative rate produces patterns (Figures 8(a) and 10 (a)) similar to that of the gold standard data (Figure 6). For the Y2H yeast protein interaction network (Figure 5), *S*4 gives the best approximation, but still differs slightly in the probabilities of nodes with small connectivity. This suggests that the real error structure of the Y2H analyses may be more complicated than all the simple proposals we have considered.

## Conclusion

This article first investigates the impact of erroneous links on network topological inference. From our theoretical and simulation results, we find that, under a simple error mechanism, the scale-free property is preserved for moderate connectivities. But the linear pattern is distorted at both the small and large connectivity regions. Accordingly, we recommend to use robust estimators (e.g. LTS) that are more resistant to the outliers at both ends of the distribution to estimate the scale parameter *γ*.

Moreover, we have also explored possible error mechanisms of the yeast protein interaction data by simulations considering nine different error mechanisms. The results suggest that changes in the overall false positive rates have little impact on the resulting connectivity distribution, but increasing the overall false negative rates can increase the probability of nodes with small connectivities under some error mechanisms, and hence decrease the scale parameter *γ*. The connectivity distribution can be very stable under several error mechanisms when the overall false positive rates and false negative rates change, which suggests that in certain situations the observed data can provide suffcient topological information on the underlying network structure even when the false negative rates are quite high.

The simple error mechanism that assumes that the false positive rate and false negative rate of each protein pair are constants agrees well with the MIPS gold standard data when the false negative rate is high. A different error mechanism is suggested for the Y2H data, where more connected protein pairs tend to have higher false positive rates and lower false negative rates. As this error mechanism provides only a reasonable approximation to the Y2H data, more sophisticated mechanisms might be needed to better capture its error structure.

## Methods

### Preferential attachment growth model

In a series of papers [15,16], Barabási *et al*. demonstrated that a scale-free network could be obtained by growing from a small number of isolated nodes by preferential attachment. The simulation scheme is defined in two steps:

1. *Growth*: starting with a small number (*m*_0_) of nodes, add a new node at every time step and connect it to *m *(≤ *m*_0_) nodes already present in the system

2. *Preferential attachment*: The new node is more likely to connect to nodes with larger connectivity. The probability Π_*i *_that a new node will be connected to node *i *depends on its connectivity *k*_*i*_, such that .

### Least Trimmed Squares (LTS)

The basic idea of LTS estimation is to minimize the sum of *h *smallest squared residuals instead of all squared residuals in the OLS to achieve robustness and also maintain good effciency. Please refer to [9] for more details of the algorithm, such as practical choices of *h*. In this article, the LTS estimation is performed using the lqs() function implemented in R [17].

## Authors' contributions

HZ had the initial idea and initiated the study. NL conducted the data analyses, and created all tables and figures, under the supervision of HZ. Both authors read and approved the final manuscript.

## Supplementary Material

Additional File 1 Tables of the estimates of the scale parameter *γ*. Click here for file

## Figures and Tables

**Table 1:** Nine error mechanisms.

Error mechanism	*p*_*ij*_	*q*_*ij*_
*S*1	constant	constant
*S*2	constant	increasing
*S*3	constant	decreasing
*S*4	increasing	constant
*S*5	increasing	increasing
*S*6	increasing	decreasing
*S*7	decreasing	constant
*S*8	decreasing	increasing
*S*9	decreasing	decreasing

**Table 2:** Parameter estimates for *Net*_0_.

Parameter	OLS	M-estimation	LTS
log *c*	1.4600	1.7846	4.008
*γ*	2.0918	2.1769	2.803
