# Supplementary material for: Are scale-free networks robust to measurement errors?
Source: BMC Bioinformatics. 2005 May 16;6:119. doi: 10.1186/1471-2105-6-119 (PMC1156868; doi:10.1186/1471-2105-6-119)
Supplement: Additional File 1 — Tables of the estimates of the scale parameter γ. [file 1471-2105-6-119-S1.pdf]

TABLES

Table **A.** 1: OLS estimates of γ of perturbed scale-free network of size 1000 under different false positive rates and false negative rates. Numbers in parentheses are standard errors of the associated estimates. The estimates are obtained after removing the outliers at the tail of the connectivity distributions.

	r_{FP}								
r_{FN}	0.1	0.2	0.3	0.4	0.5	0.6	0.7	0.8	0.9
0.000100	2.6585	2.7834	2.8723	2.9294	2.9870	3.0502	3.1273	3.2701	3.5037
	(.0546)	(.0475)	(.0392)	(.0338)	(.0290)	(.0248)	(.0231)	(.0315)	(.0580)
0.000125	2.6614	2.7870	2.8765	2.9346	2.9935	3.0586	3.1383	3.2856	3.5334
	(.0545)	(.0475)	(.0392)	(.0340)	(.0294)	(.0257)	(.0248)	(.0337)	(.0629)
0.000150	2.6642	2.7905	2.8806	2.9398	2.9999	3.0667	3.1491	3.3007	3.5617
	(.0545)	(.0475)	(.0394)	(.0343)	(.0300)	(.0267)	(.0266)	(.0359)	(.0675)
0.000175	2.6669	2.7939	2.8847	2.9448	3.0061	3.0747	3.1595	3.3153	3.5889
	(.0545)	(.0476)	(.0395)	(.0346)	(.0305)	(.0277)	(.0284)	(.0382)	(.0718)
0.000200	2.6697	2.7973	2.8886	2.9497	3.0123	3.0825	3.1697	3.3295	3.6150
	(.0545)	(.0478)	(.0397)	(.0350)	(.0312)	(.0289)	(.0302)	(.0404)	(.0759)
0.000225	2.6723	2.8006	2.8925	2.9545	3.0183	3.0901	3.1797	3.3433	3.6401
	(.0546)	(.0479)	(.0400)	(.0354)	(.0319)	(.0300)	(.0320)	(.0427)	(.0799)
0.000250	2.6749	2.8039	2.8964	2.9592	3.0242	3.0975	3.1895	3.3568	3.6643
	(.0546)	(.0481)	(.0402)	(.0359)	(.0327)	(.0313)	(.0339)	(.0449)	(.0837)
0.000275	2.6775	2.8071	2.9001	2.9639	3.0300	3.1049	3.1990	3.3699	3.6877
	(.0547)	(.0483)	(.0405)	(.0364)	(.0335)	(.0325)	(.0357)	(.0471)	(.0874)
0.000300	2.6800	2.8102	2.9038	2.9684	3.0356	3.1120	3.2083	3.3827	3.7103
	(.0548)	(.0485)	(.0409)	(.0369)	(.0343)	(.0338)	(.0375)	(.0493)	(.0909)

Table **A.** 2: OLS estimates of γ of perturbed scale-free network of size 7000 under different false positive rates and false negative rates. Numbers in parentheses are standard errors of the associated estimates. The estimates are obtained after removing the outliers at the tail of the connectivity distributions.

	r_{FP}								
r_{FN}	0.1	0.2	0.3	0.4	0.5	0.6	0.7	0.8	0.9
0.000100	2.8062	2.9008	2.9677	3.0210	3.0739	3.1355	3.2145	3.3284	3.5799
	(.0466)	(.0411)	(.0353)	(.0340)	(.0321)	(.0354)	(.0431)	(.0586)	(.0970)
0.000125	2.8139	2.9107	2.9797	3.0358	3.0914	3.1573	3.2423	3.3651	3.6360
	(.0482)	(.0435)	(.0384)	(.0380)	(.0367)	(.0410)	(.0496)	(.0659)	(.1061)
0.000150	2.8206	2.9195	2.9902	3.0490	3.1070	3.1768	3.2671	3.3976	3.6842
	(.0501)	(.0462)	(.0417)	(.0422)	(.0414)	(.0465)	(.0559)	(.0730)	(.1152)
0.000175	2.8264	2.9271	2.9996	3.0607	3.1210	3.1942	3.2891	3.4262	3.7256
	(.0522)	(.0492)	(.0452)	(.0465)	(.0461)	(.0520)	(.0621)	(.0799)	(.1238)
0.000200	2.8313	2.9337	3.0078	3.0710	3.1334	3.2097	3.3085	3.4514	3.7614
	(.0546)	(.0524)	(.0488)	(.0508)	(.0508)	(.0573)	(.0680)	(.0865)	(.1318)
0.000225	2.8354	2.9393	3.0149	3.0800	3.1443	3.2233	3.3257	3.4735	3.7927
	(.0572)	(.0557)	(.0525)	(.0552)	(.0554)	(.0625)	(.0738)	(.0928)	(.1393)
0.000250	2.8387	2.9440	3.0211	3.0879	3.1539	3.2353	3.3408	3.4929	3.8202
	(.0599)	(.0592)	(.0563)	(.0595)	(.0599)	(.0675)	(.0793)	(.0988)	(.1462)
0.000275	2.8413	2.9479	3.0263	3.0946	3.1622	3.2458	3.3541	3.5101	3.8444
	(.0627)	(.0626)	(.0600)	(.0638)	(.0644)	(.0724)	(.0847)	(.1046)	(.1529)
0.000300	2.8431	2.9509	3.0306	3.1003	3.1694	3.2549	3.3657	3.5252	3.8655
	(.0656)	(.0662)	(.0638)	(.0680)	(.0687)	(.0772)	(.0898)	(.1101)	(.1594)

Table **A.** 3: LTS estimates of γ under error mechanism $S1$.

	r_{FP}								
r_{FN}	0.1	0.2	0.3	0.4	0.5	0.6	0.7	0.8	0.9
0.000100	2.9071	2.8231	2.8278	2.9118	2.9780	2.8701	3.2091	3.3813	2.2851
0.000125	2.9390	2.7974	2.9413	3.0630	3.1484	3.2308	3.2072	3.5419	3.3036
0.000150	2.9004	2.9686	2.8436	3.0960	3.1125	2.9856	3.4359	2.7048	3.8429
0.000175	2.9361	2.8284	3.0388	2.9116	3.1243	2.9213	3.4873	3.4162	2.1191
0.000200	2.7906	2.8346	2.8987	2.9988	3.1465	3.1178	3.1057	3.6301	3.6675
0.000225	2.9031	2.8335	2.9763	3.1707	3.2659	3.1326	3.4843	3.7824	4.0427
0.000250	2.8702	2.8046	3.1654	3.2297	1.1651	3.2840	2.5065	1.5409	2.7901
0.000275	2.8339	3.1851	3.0857	3.1298	3.0586	3.1076	3.3487	3.5856	4.1232
0.000300	2.9122	2.8934	3.2199	2.9939	3.2147	3.2930	3.4902	3.9753	4.3546

Table **A.** 4: LTS estimates of γ under error mechanism $S3$.

	r_{FP}								
r_{FN}	0.1	0.2	0.3	0.4	0.5	0.6	0.7	0.8	0.9
0.000100	2.5508	2.5108	2.3465	2.1786	2.3493	2.0614	1.8480	1.5165	1.6383
0.000125	2.8005	2.7202	2.5123	2.3124	2.1253	2.0212	1.8891	1.8786	1.6214
0.000150	2.7020	2.5364	2.4164	2.0453	2.0856	1.8110	1.9724	1.4755	1.5361
0.000175	2.5586	2.6510	2.4405	2.2310	2.2180	2.0738	1.8628	1.6593	1.6490
0.000200	2.5850	2.5959	2.5744	2.2597	2.3324	2.0856	1.9280	1.6682	1.7270
0.000225	2.5069	2.7610	2.3541	2.2500	2.2386	2.1360	1.9451	1.6821	1.6066
0.000250	2.5235	2.5139	2.2902	2.1961	2.0886	2.0867	1.8267	1.6236	1.3869
0.000275	2.6290	2.5059	2.3913	2.3150	2.1890	2.1923	1.5604	1.6361	1.6231
0.000300	2.5741	2.5008	2.3740	2.3609	2.0663	2.0281	1.9791	1.5099	1.7484

Table **A.** 5: LTS estimates of γ under error mechanism $S4$.

	r_{FP}								
r_{FN}	0.1	0.2	0.3	0.4	0.5	0.6	0.7	0.8	0.9
0.000100	2.6735	2.6337	2.7786	2.7938	2.6175	2.7846	2.6067	2.7397	2.8142
0.000125	2.6224	2.9426	2.7963	2.6639	2.9261	2.8504	3.0650	2.8934	2.6921
0.000150	2.9173	2.9498	2.7133	2.8139	3.0014	2.6427	2.9280	2.8628	2.8690
0.000175	2.6874	2.6680	2.7788	2.8274	2.6624	2.8934	2.9042	2.7492	2.7427
0.000200	2.8149	2.6307	2.9737	2.8376	2.6218	2.9884	2.8830	2.8694	2.9577
0.000225	3.0139	2.7138	2.9863	2.6903	2.8760	2.9589	2.8148	3.0004	2.8954
0.000250	2.9113	3.0340	2.9966	2.6749	2.9313	2.8546	2.6744	2.7588	2.8207
0.000275	2.8980	2.7047	2.6394	2.8546	2.7945	2.8026	2.6700	2.8006	2.7025
0.000300	2.9241	2.9129	3.0583	2.8345	2.8166	2.6056	2.6838	2.6837	2.6675

Table **A.** 6: LTS estimates of γ under error mechanism $S7$.

	r_{FP}								
r_{FN}	0.1	0.2	0.3	0.4	0.5	0.6	0.7	0.8	0.9
0.000100	3.0706	2.6970	2.8494	2.7277	3.0724	2.7234	3.1850	2.8647	3.0582
0.000125	3.0671	2.9249	3.0927	2.8832	2.9215	3.0218	3.1551	3.0275	3.0009
0.000150	3.0675	2.9322	2.9954	2.9862	2.7633	2.9474	2.8864	2.7652	2.9206
0.000175	3.0162	3.1246	3.0656	3.1851	3.0555	2.9096	2.9406	2.8944	3.0687
0.000200	3.0370	3.1561	3.1284	3.1514	2.7934	3.3103	2.7741	3.1774	3.1540
0.000225	3.0893	3.1791	3.1892	3.1153	3.1791	3.0718	3.0136	3.1314	2.8911
0.000250	3.0748	3.1593	3.2057	3.1911	2.6101	3.0482	2.8106	3.1605	2.8149
0.000275	3.0730	3.1423	3.0090	3.1833	3.2136	3.1695	2.9879	2.9612	3.1797
0.000300	2.9575	2.9793	3.2462	3.2165	2.5594	3.3011	3.2269	3.0794	3.2350

Table **A.** 7: LTS estimates of γ under error mechanism $S8$.

	r_{FP}								
r_{FN}	0.1	0.2	0.3	0.4	0.5	0.6	0.7	0.8	0.9
0.000100	2.7952	2.6632	2.5882	2.3119	2.2716	2.1205	1.7567	1.8561	1.4607
0.000125	2.8543	2.7358	2.5129	2.2666	2.1613	2.3507	1.8441	2.2466	1.4802
0.000150	2.7836	2.8190	2.5096	2.2949	2.4069	2.0378	1.9993	1.7510	1.4999
0.000175	2.7254	2.5517	2.5203	2.4492	2.4752	2.0918	1.9961	1.5665	1.6622
0.000200	2.9648	2.7834	2.6126	2.6724	2.1898	1.9879	1.9967	1.9449	1.7226
0.000225	2.7409	2.6515	2.5121	2.4366	2.2638	2.1726	1.7585	1.7566	1.8886
0.000250	2.7047	2.4795	2.4292	2.4886	2.2815	1.9711	1.9981	1.7653	1.8662
0.000275	2.6774	2.5110	2.5926	2.4269	2.3487	2.2551	1.9938	2.2469	1.8629
0.000300	2.7317	2.7426	2.5718	2.3722	2.3155	2.2054	1.9623	1.7174	1.8319
